# Mark Twain’s Receipt for New England Pie

**Published:** 1880-12

**Authors:** 


Mark Twain's Recipe for New England Pie.—To make this excellent breakfast dish proceed as follows: Take a sufficiency of water and a sufficiency of flour and construct a bullet-proof dough. Work this into the form of a disk, with the edges turned up some three-fourths of an inch. Toughen and kiln-dry it a couple of days in a mild but unvarying temperature. Construct a cover for this redoubt in the same way and of the same material. Fill with stewed dried apples; aggravate with cloves, lemon-peel, and slabs of citron; add two portions of New Orleans sugar; then solder on the lid and set in a safe place until it petrifies. Serve cold at breakfast and invite your enemy.—*Louisville Medical News.*

